# Effects of Helicopter Parenting on Tutoring Engagement and Continued Attendance at Cram Schools

**DOI:** 10.3389/fpsyg.2022.880894

**Published:** 2022-04-13

**Authors:** Ya-Jiuan Ho, Jon-Chao Hong, Jian-Hong Ye, Po-Hsi Chen, Liang-Ping Ma, Yu-Ju Chang Lee

**Affiliations:** ^1^Taipei City Government, Taipei, Taiwan; ^2^Department of Industrial Education, National Taiwan Normal University, Taipei, Taiwan; ^3^Chinese Language and Technology Center in Learning Sciences, National Taiwan Normal University, Taipei, Taiwan; ^4^Faculty of Education, Beijing Normal University, Beijing, China; ^5^Department of Educational Psychology and Counseling, National Taiwan Normal University, Taipei, Taiwan; ^6^Department of Chinese as a Second Language, National Taiwan Normal University, Taipei, Taiwan; ^7^Executive Master of Business Administration, National Taiwan Normal University, Taipei, Taiwan

**Keywords:** English cram schools, helicopter parenting, learning engagement, mummy’s child, tutoring

## Abstract

Attending cram school has long been a trend in ethnic Chinese culture areas, including Taiwan. Despite the fact that school reform policies have been implemented in Taiwan, cram schools have continued to prosper. Therefore, in this educational culture, how to achieve a good educational effect is also a topic worthy of discussion. However, whether students really engage in those tutoring programs provided by cram schools has seldom been studied. To address this gap, this study explored how parents’ hovering attitude toward life and coursework influences their children’s engagement in cram schools. This study targeted those students who attend English cram schools to test the correlates between two types of helicopter parenting, tutoring engagement and continued attendance at cram schools. A total of 320 questionnaires were sent out, and 300 were returned, giving an overall response rate of 93.75%. Excluding seven incomplete or invalid questionnaires, 293 valid questionnaires were received. The results of this study show that hovering behavior awareness is negatively related to cram school engagement, whereas cram school engagement is positively related to the intention to continue attending cram school. Moreover, the results imply that parents should alleviate their helicoptering behavior to enhance their children’s engagement in cram school tutoring programs.

## Introduction

In order to improve students’ performance in academic subjects (Zhang et al., 2021), profit-oriented individuals or school-like organizations, specialized schools, or so-called cram schools, offer extra-curricular instruction to students. Their curricula mimic the mainstream school curriculum but they differ from the mainstream system in their instruction. Because of this, cram schools are referred to as “shadow education.” Cram schooling is prevalent worldwide (Yung, 2020a,b) and has been given different names in different countries, such as Buxiban in Taiwan, Juku in Japan, Hagwon in Korea, and private tuition or the shadow education system in Western countries. What cram schooling does is train students’ ability of taking tests on academic subjects in order to pass the entrance examinations of better schools (Wang and Wu, 2021). One of students’ popular out-of-school learning activities during the past few decades has been to go to cram schools, and it is believed that a great number of students around the world have received some type of cram schooling (Liu, 2012). According to Bray and Lykins (2012), more than half of the secondary students in Asian countries, for example, China, Japan, South Korea, and Thailand, go to cram schools for tutoring, and the numbers in Western countries are growing fast as well (e.g., Stastný, 2016; Pearce et al., 2018). In cram schools, students passively absorb “pre-processed” information and then “regurgitate” it in school examinations (Bray and Lykins, 2012). However, there is little research on students’ perceptions of the learning environment that affects their engagement, which is key to learning (Diseth et al., 2010). Central to the learning environment in cram schools is the expectation that the more students invest time and effort in educationally purposeful tasks, the more they will gain from their learning experience (Price et al., 2011). In this sense, in order to understand the learning effectiveness of these schools, it is important to study engagement in cram school environments. Therefore, the present study explored the tutoring engagement of cram school attendees.

As cram school instruction may focus on school content with the hope of improving students’ academic performance through relatively short, temporary instruction (Wang and Wang, 2021), for example, in many English-learning cram schools that offer English tutoring classes are mainly intent on increasing students’ achievement in mainstream education and on high-stakes examinations (Bray, 2011; Yung and Chiu, 2020). In Taiwan, Chung (2013) surveyed 365 senior high school students regarding their motivation to learn English and their receipt of tutoring from cram schools, of whom 342 reported that undertaking tutoring from cram schools, meaning investing time and putting extra effort into learning activities, is beneficial for obtaining high examination scores, which leads to an increase in academic performance. However, his paper contextualizes the discussion in sociocultural conditions, focusing on the Taiwanese setting; little research has collected data related to the role of students’ engagement in the effectiveness of English tutoring. Thus, this study aimed to understand the effects of English tutoring engagement at Taiwan’s cram schools.

Cram schools have diversified their breadth to coordinate with recent educational reforms. In Taiwan, in order for students to learn new skills, they have started to attend cram schools even earlier to have a better chance to apply for the new multi-phased entrance program (providing alternative methods for entrance into senior high schools and universities) (Liu, 2012; Lo and Lin, 2020). A previous study indicated that parents’ attitudes will influence whether they assist their children in doing homework or send them to be tutored at cram schools (Chang, 2019). This centralization of parenting style has indirectly created the current emphasis on cram schools. Consequently, how parenting style affects children’s behavior in cram schools deserves study. This study therefore investigated how helicopter-type parenting affects children’s engagement in English cram school tutoring.

Based on the theory of control-value of achievement emotions that Pekrun proposed in 2006. It is an emotion associated with academic achievement activities and their successful and unsuccessful outcomes (Camacho-Morles et al., 2021). There are two types of outcome emotions, perspective outcome emotions related to whether success can be achieved or failure can be avoided; and retrospective outcome emotions, meaning whether oneself or external sources such as another person or the environment has an effect on the outcome. for example, timeframe for engagement. Retrospective outcome emotions assume that parents opt for encouraging or controlling their children’s attendance of private tutoring if the expectation is to reach a desired educational goal (Guill and Lintorf, 2019). Parents might weigh up whether their child’s study behavior is sufficient, and they might try to encourage their child to receive tutoring (Kuan, 2011). On the other hand, activity-related emotions suppose that students have an intention to engage in the totality of each interaction and control themselves “on-task,” and can deepen our understanding of the learning content in private tutoring programs (Pomerantz, 2019). However, based on activity-related emotion, how students engage in private English tutoring has not been extensively studied; thus, this study took activity-related emotion to form a conceptual model to explore the correlates between parenting style, students’ tutoring engagement, and continued attendance at cram schools. Based on the research objectives, this study proposes 9 research hypotheses and elicits 2 research questions containing the following:

RQ1: Are life and coursework hovering negatively related to three types of tutoring engagement?
RQ2: Are three types of tutoring engagement positively related to continued attendance?

### Perceived Helicopter Parenting and Its Effects

A lack of the proper transitions in the parent-child relationship as children develop can limit emerging adults from proceeding through this stage during which they explore the world (Arnett, 2000). Previous studies have indicated that the expectations placed on parents are increasing, and helicopter parenting is becoming more widespread. “Middle-class circumstances and resources” is what is causing the intensive parenting urge (Chudacoff, 2007; Granja et al., 2015) and even those with ample resources for their children may struggle to adhere to an intensive parenting style (Valentine et al., 2019) or helicopter parenting (Padilla-Walker and Nelson, 2012). Helicopter parenting is a relatively new phenomenon that describes a specific type of overparenting (Dumont, 2021) which involves continuous control by the parent over the child’s life, from daily life to interpersonal relationships, and which may hinder the child’s efforts to satisfy their desire for autonomy (Carr et al., 2021). Helicopter parents hover over their children’s lives by being overly protective and unwilling to let go (van Ingen et al., 2015). Low tolerance for error and high parental expectations in authoritarian parenting may be more likely to cause children to internalize similar standards of evaluation into their own performance (Chen et al., in press). Padilla-Walker and Nelson (2012) argued that helicopter parents try to structure their children’s behavioral world including their daily activities and coursework in ways that are intrusive and manipulative of their children’s thoughts, feelings, and attachment to their parents. Accordingly, this study explored the consequences of parents’ actions in guiding the participants’ behavior through adopting life hovering and coursework hovering behaviors.

### Tutoring Engagement

Behavioral (e.g., completion of academic tasks, on-task behavior), emotional (e.g., excitement, enjoyment in learning activities), and cognitive (e.g., mental effort to understand complex ideas) ways are the connections between students and learning, which is also known as learning engagement (Fredricks et al., 2004; Lawson and Lawson, 2013). As McCormick et al. (2013) pointed out, a substantial amount of research shows that time on task and quality of effort are central to students’ learning and achievement, and are relevant to student engagement (e.g., Ryu and Lombardi, 2015; Sinatra et al., 2015). To evaluate students’ engagement, person-centered approaches may allow for the examination of profiles characterized by different configurations of engagement by individuals (Bae and DeBusk-Lane, 2019). For example, for those students who are excited to participate in learning but who may not have been engaged in the learning activities for processing deep understanding of the content, it is possible that they may receive the results from the examination of a high level of emotional engagement combined with a low level of cognitive engagement. Students who may perform well on on-task behaviors, but who lack concentration in their learning are examples of high behavioral engagement with low cognitive engagement (Bae and DeBusk-Lane, 2019). Taking a person-centered approach to examine how students vary in their multivariate engagement profiles in cram school tutoring is not often seen in previous research; thus, the present study explored participants’ three types of tutoring engagement in English cram schools.

### Continued Attendance at English Cram Schools

Emotions regarding attitudes, motivation, affect, interests, and goal orientations are the wide range of disparate constructs in secondary language learning (e.g., Gardner and MacIntyre, 1993; Bown and White, 2010). A previous study has shown that motivational purposes in educational settings with the use of positive psychology interventions (PPI) have striking efficiency (Muro et al., 2018). Moreover, they explained what PPI does to develop students’ cognitive, emotional, and behavioral engagement to increase motivation for continued learning and so improve academic outcomes (Muro et al., 2018). As students learn and perceive that they are becoming proficient in certain subjects, it could prompt them to continue learning and improve their academic outcomes as a result. In other words, students’ motivation influences what and how they learn (Muro et al., 2018). Dewaele (2005) argued that positive intervention with pleasure while performing L2 learning tasks can signal arousal of the brain limbic system and influence continuance of learning. Accordingly, the present study explored participants’ continued attendance at English cram schools (hereafter, continued attendance).

### Research Model

**FIGURE 1:**
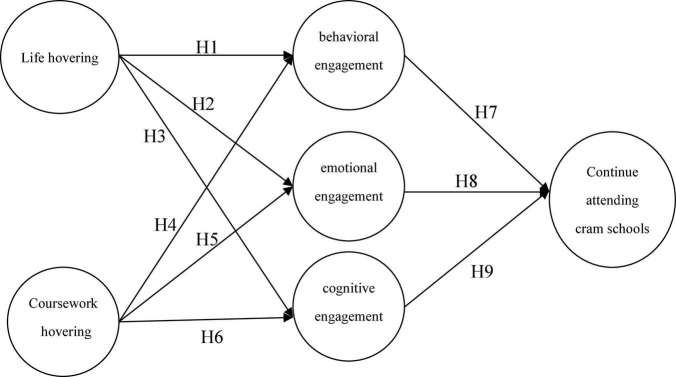
Research model.

Increasing grades and learning achievement as the congregation of adaptive outcomes is related to engagement (King and Gaerlan, 2014) and also improved attendance and retention (Garn et al., 2017). Accordingly, the research model is proposed as follows. Increasing grades and learning achievement as the congregation of adaptive outcomes is related to engagement (King and Gaerlan, 2014) and also improved attendance and retention (Garn et al., 2017). Accordingly, the research model is proposed as follows, as shown in Figure 1.

### Research Hypotheses

#### Linkage Between Helicopter Parenting and Tutoring Engagement

How parents are responsive and involved in their children’s life or school-related activities is considered as the degree of parental involvement (Luo et al., 2016). Furthermore, parental involvement has an impact on students’ motivation and emotion in learning. For instance, students’ expectancy and value beliefs in doing homework are influenced by how their parents react to their learning (Trautwein et al., 2006). One study has shown that exhibiting lower homework procrastination and higher effort and achievement can be predicted by having parents who are involved in guiding their children to do homework (Dumont et al., 2014). Private tutoring seems to contribute to continued education; if parents see an educational future for their children, they are likely to support their children’s private tutoring attendance (Chugh, 2011; Bray, 2017). Despite serious theoretical advances in the role of engagement in academic settings (Pekrun and Stephens, 2010), the relationship among learning engagement and its antecedent factors has seldom been shown with empirical evidence (Goetz et al., 2010). To address helicopter parenting as an antecedent in the relation with learning engagement, the present study examined how participants’ perceptions of being helicopter parented in terms of life hovering and coursework hovering affected their appraisal of tutoring engagement. Thus, the following hypotheses were proposed:

H1: Life hovering is negatively related to behavioral engagement.
H2: Life hovering is negatively related to emotional engagement.
H3: Life hovering is negatively related to cognitive engagement.
H4: Coursework hovering is negatively related to behavioral engagement.
H5: Coursework hovering is negatively related to emotional engagement.
H6: Coursework hovering is negatively related to cognitive engagement.

#### The Relevance of Tutoring Engagement to Continued Attendance

Previous studies indicate that practice plays an important role in learning, since it may be able to improve the learning process (Gherardi, 2018). Such practice in cram school may help students solve problems, resulting in better engagement (Marques, 2019). For example, engagement in cram school was found to help students in Taiwan with their analytic ability and mathematics scores (Liu, 2012). Private tutoring may also be a double-edged sword (Han and Lee, 2016); practicing may result in students concentrating just on superficial indicators, neglecting deep learning (Sabbagh et al., 2017). Briefly, engagement may be differentially associated with student outcomes (Bae and DeBusk-Lane, 2019). Previous studies have indicated the relevance between cram schooling and improvements in academic performance; however, others have not identified a consequential relationship (Lee et al., 2010; Liu, 2012). Despite those studies of engagement influencing the learning outcomes of the private tutoring system, one study indicated the importance of motivation in having private tutoring impact the possible continuance mechanism to attend English cram schools (Chang, 2019). Thus, how participants’ tutoring engagement is related to their continued attendance was hypothesized as follows:

H7: Behavioral engagement is positively related to continued attendance.
H8: Emotional engagement is positively related to continued attendance.
H9: Cognitive engagement is positively related to continued attendance.

### Procedure

Purposive sampling was adopted in this study. The target samples were from English cram schools located in northern Taiwan. With respect to family socioeconomic status, most middle-class parents who work in white-collar jobs tend to send their children to English cram schools to ensure that they have the advanced English ability necessary to perform well in future high-stakes examinations (Chang, 2019). Thus, the participants of this study were students of eight English cram schools. The questionnaire was delivered to 320 attendees of these cram schools. The sampling period was from January 2019 to February 2019. There 300 were returned, giving an overall response rate of 93.75%. Excluding seven incomplete or invalid questionnaires, 293 valid questionnaires were received. To adhere to ethical standards, students were provided with information about what they were being asked to do, their consent was requested, and they were given the option of withdrawing from the study if they so wished.

### Participants

Weston and Gore (2006) recommend a minimum sample size of 200 for any SEM study, and the effective participants in this study were above the recommended standard. Valid responses were received from 293 participants in this research. The gender distribution of the study sample was 49.8% female and 50.2% male students. Regarding age, 37.9% were seventh graders, 36.7% were eighth graders, and 25.4% were ninth graders. The weekly frequency with which each participant attended cram schools was: less than two times (3.4%), twice (10.2%), three times (19.1%), four times (26.3%), five times (18.1%), and six or more times (22.9%). Most participants attended cram schools four times per week.

### Measurement

The questionnaire and scale used in this research were translated and edited based on previous studies, and were subject to expert validity review by three domain experts. After the content validity was confirmed, we invited seven junior high school students who also attended English cram schools to answer the questionnaire. Based on their feedback, a revised version of the questionnaire with face validity was produced. After its expert validity was confirmed, the questionnaire was then piloted by another 10 students to confirm that they understood the meanings of the items. A 5-point Likert scale with the following options was adopted: 1-*strongly disagree*; 2-*disagree*; 3-*undecided*; 4-*agree;* and 5-*strongly agree*. Based on the confirmatory research, the reliability and validity of the questionnaire were re-tested after data collection.

#### Helicopter Parenting

Hong et al. (2015) differentiated helicopter parenting into life hovering and coursework hovering. The scale of this study used the content of the questionnaire compiled by Hong et al. (2015) to measure the participants’ perceptions of being subject to helicopter parenting. Eight items related to life hovering, and six items related to coursework were included, as shown in Table A1. This scale has α = 0.80, CR = 0.86, AVE = 0.56, and FL = 0.68∼0.81.

#### Tutoring Engagement

Fredricks et al. (2004) divided learning engagement into three dimensions: behavioral, emotional and cognitive learning engagement. Based on this differentiation method, this study referred to the concept stated by Luan et al., 2020) to measure the participants’ perceptions of being subject to tutoring engagement. Each construct of engagement contained seven items, as shown in Table A1. This scale has α = 0.83∼0.86, CR = 0.80∼0.86, AVE = 0.51∼0.61, and FL = 0.70∼78.

#### Continued Attendance

Continuity is a form of post-adoption behavior (Chang, 2013). This study utilized and revised the continuous participation willingness scale developed by Hong et al. (2014) to measure participants’ perceptions of continued attendance. Eight items were included in this construct, as shown in Table A1. This scale has α = 0.90, AVE = 0.62, CR = 0.87, and FL = 0.65∼0.90.

## Results and Discussion

In this study, SPSS 23.0 was used for reliability and validity analysis, and AMOS 20.0 was used for item analysis, model fit analysis and path analysis. The relevant analysis criteria and results are as the following:

### Item Analysis

In this phase, 293 participants were used for item analysis in this study. Next, the item analysis method used in this study was first-order confirmatory factor analysis (CFA). Scholars suggest that the value of χ^2^ / df should be less than 5; thus, the root mean square error of approximation (RMSEA) of the obtained result should be less than 0.10. As the goodness of fit index (GFI) and adjusted goodness of fit index (AGFI) should be larger than 0.80, items with a factor loading (FL) of less than 0.50 should be deleted from the original questionnaire (Hair et al., 2010; Kenny et al., 2015). The deletion results of each section are as follows: Life hovering: from eight items to six; Coursework hovering: from six items to five; Behavioral engagement: from seven items to four; Emotional engagement: from seven items to five; Cognitive engagement: from seven items to five; and Continued attendance at cram school: from eight items to five, as shown in Table 1.

**TABLE 1:** Item analysis.

Index	χ ^2^/df	RMSEA	GFI	AGFI	FL	*t*
Threshold	<5	<0.10	>0.80	>0.80	>0.50	>3
Life hovering	1.39	0.04	0.98	0.96	0.55∼0.90	16.29∼21.17
Coursework hovering	1.68	0.05	0.99	0.97	0.76∼0.91	16.84∼23.98
Behavioral engagement	2.20	0.06	0.99	0.96	0.80∼0.90	20.49∼24.30
Emotional engagement	2.57	0.07	0.97	0.94	0.76∼0.82	17.55∼20.31
Cognitive engagement	2.32	0.07	0.99	0.96	0.62∼0.84	16.20∼21.20
Continue attending	2.52	0.07	0.97	0.94	0.81∼0.88	20.37∼24.22

The external validity of the item can be determined by the critical ratio between the top and bottom groups (Cor, 2016), that is the top 27% and the bottom 27% of all respondents’ values of each item were used to perform a *t* test. If the *t*-value (critical ratio) is larger than 3 (^***^p < 0.001), the item is considered to have good external validity. Table 2 shows that the *t*-value is larger than 16.20 (^***^p < 0.001), which means that all of the items retained in the questionnaires are discriminative. All of them are able to verify the response level of different samples (Green and Salkind, 2004), as shown in Table 1.

**TABLE 2:** Reliability and validity analysis.

Construct	*M*	*SD*	α	CR	FL	AVE
Threshold	–	–	>0.70	>0.70	>0.50	>0.50
Life hovering	2.30	0.78	0.94	0.94	0.85	0.73
Coursework hovering	2.07	0.81	0.94	0.94	0.87	0.76
Behavioral engagement	3.75	0.84	0.91	0.90	0.84	0.70
Emotional engagement	3.44	0.68	0.91	0.91	0.79	0.62
Cognitive engagement	3.52	0.71	0.87	0.86	0.74	0.56
Continued attendance	3.63	0.78	0.94	0.93	0.83	0.69

### Reliability and Validity Analysis

In this phase, 293 participants were used for item analysis in reliability and validity study, and a Cronbach’s α test was used to confirm the internal consistency of the analysis dimensions, and the reliability was retested with composite reliability (CR). Scholars suggest that values should be considered acceptable when the Cronbach’s α value is larger than 0.70 (Hair et al., 2010). Hair et al. (2010) suggested that the CR value should be larger than 0.70 to be considered as having composite reliability. In this study, the Cronbach’s α value is between 0.87 and 0.94 (as shown in Table 2), conforming with the suggested thresholds.

The convergence validity of this study was determined by the factor loading (FL) and the average variance extracted (AVE). Hair et al. (2010) suggested that the FL value should be larger than 0.50 to have convergence validity. Thus, items with values of less than 0.50 should be deleted. In this study, the FL values is between 0.74 and 0.87 (as shown in Table 2). Hair et al. (2011) suggested that the AVE value of a dimension should be larger than 0.50 to have convergence validity. In this study, the AVE value is between 0.56 and 0.76 (as shown in Table 2).

The discriminative validity is determined if the AVE root value of a construct is greater than the Pearson correlation coefficient value of another construct (Awang, 2015). All constructs have good discriminative validity, as shown in Table 3.

**TABLE 3:** Construct discriminative validity analysis.

Construct	1	2	3	4	5	6
(1) Life hovering	(0.85)					
(2) Coursework hovering	0.53	(0.87)				
(3) Behavioral engagement	–0.34	–0.34	(0.84)			
(4) Emotional engagement	–0.33	–0.33	0.39	(0.79)		
(5) Cognitive engagement	–0.32	–0.35	0.58	0.67	(0.75)	
(6) Continue attending	–0.26	–0.28	0.49	0.57	0.61	(0.83)

### Model Fit Analysis

In this phase, 293 participants were used for item analysis in model fit analysis. Hair et al. (2010) suggested that the value of the chi-square degree of freedom ratio (χ^2^ / df) should be less than 5. The RMSEA should be less than 1. The value of GFI, AGFI, normed fit index (NFI), non-normed fit index (NNFI), comparative fit index (CFI), incremental fit index (IFI), and relative fit index (RFI) should all be larger than 0.8 (Abedi et al., 2015). As for the parsimonious normed fit index (PNFI) and parsimonious goodness of fit index (PGFI), the value should be larger than 0.5 (Hair et al., 2010). The fitting index values of this study are as follows: χ^2^ = 923.92, df = 455, χ^2^/df = 2.03, RMSEA = 0.060, GFI = 0.843, AGFI = 0.82, NFI = 0.885, NNFI = 0.93, CFI = 0.94, IFI = 0.94, RFI = 0.97, PNFI = 0.81, and PGFI = 0.73. Each fitting index value in this study meets the suggested standards, and has a good model fit.

### Research Model Verification

**FIGURE 2:**
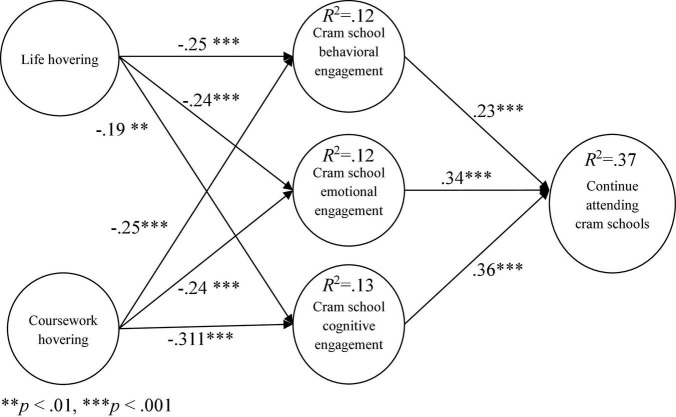
Verification of the research model. ^**^p < 0.01, ^***^p < 0.001.

In this study, structural equation modeling was used for model validation, and all nine research hypotheses were supported. The results of the research model verification are as follows: Life hovering awareness has negative impacts on behavioral engagement (β = −0.25^***^; *t* = −3.32), emotional engagement (β = −0.24^***^; *t* = −3.32), and cognitive engagement (β = −0.19^**^; *t* = −2.56). Coursework hovering also has negative impacts on each engagement dimension as follows: behavioral engagement (β = −0.25^**^; *t* = −3.41), emotional engagement (β = −0.24^***^; *t* = −3.29), and cognitive engagement (β = −0.31^***^; *t* = −4.13). Meanwhile, behavioral engagement has positive impacts on the intention to continue attending cram school (β = 0.23^***^; *t* = 3.60), as do emotional engagement (β = 0.340^***^; *t* = 4.694) and cognitive engagement (β = 0.36^***^; *t* = 4.26), as shown in Figure 2.

The explanatory power of life hovering awareness and coursework hovering awareness to behavioral engagement is 12%, to emotional engagement it is 12%, and to cognitive engagement it is 13%. The explanatory power of behavioral engagement, emotional engagement, and cognitive engagement to the intention to continue attending cram schools is 37%. On the other hand, the effect size of cram school behavioral engagement (*f*^2^) is 0.14, that of emotional engagement (*f*^2^) is 0.13, cognitive engagement (*f*^2^) is 0.16, and there is an effect size of 0.58 for the intention to continue attending cram school (*f*^2^).

### Discussion

Hovering behavior refers to helicopter parents paying too much attention to their children (Hong et al., 2015; Hwang et al., 2022). Participants in this study had a low awareness of life hovering (*M* = 2.30, *SD* = 0.78) and coursework hovering (*M* = 2.07, *SD* = 0.81). Behavioral engagement refers to engaging in learning activities, including paying attention to academic tasks, positive behavior, and attending school. Emotional engagement refers to the emotional attitude and recognition of the school and belongingness. Cognitive engagement refers to the self-regulation method of learning and using post-cognitive strategies (Fredricks et al., 2004; El-Sayad et al., 2021). The participants in this study were well aware of cram school behavioral engagement (*M* = 3.75, *SD* = 0.84), emotional engagement (*M* = 3.44, *SD* = 0.68), and cognitive engagement (*M* = 3.52, *SD* = 0.71). As continuity is a form of post-adoption behavior (Chang, 2013), participants in this study showed positive attitude in terms of their intention to continue attending cram school (*M* = 3.6, *SD* = 0.78). The results of the study showed that life hovering had negative impacts on the three types of tutoring engagement, so H1∼H3 were negatively verified; coursework hovering had negative impacts on the three types of tutoring engagement, so H4∼H6 were also negatively verified; however, the three types of tutoring engagement had positive impacts on continued attendance, so H7∼H9 were positively verified.

Helicopter parenting is accompanied by a series of negative consequences (Casillas et al., 2021), including subjective and academic ill effects (Schiffrin et al., 2014). In addition, research suggests that strong parenting beliefs do not help children engage in structured activities (Schiffrin et al., 2015). Helicopter parenting negatively affects child development in such aspects as emotion and learning development (Kwon et al., 2017). Hong et al. (2015) confirmed that learners with higher awareness of hovering behaviors have weaker learning situations and are more prone to procrastination. Padilla-Walker and Nelson (2012) also pointed out that the more parents are involved in their children’s daily life, the less their children will engage in school, whereas Schiffrin and Liss (2017) indicated in their study that helicopter parenting correlates with poor learning motivation. In addition, past research has found that harsh parenting, a form of negative parenting, may have a negative impact on learning engagement (Zhang and Yue, 2021). From the above literature, it can be said that hovering behavior has negative impacts on cram school engagement. The results of this study display that two types of hovering behavior awareness are negatively correlated with three kinds of English cram school engagement (behavioral, emotional, and cognitive).

Engagement is the act of meeting internal and external expectations (Guthrie et al., 2013). In recognition of the importance of student engagement to the current and future success of students (Cents-Boonstra et al., 2021). Attending cram schools might help solve students’ problems and further increase the level of their engagement (Marques, 2019). Researchers have also pointed out that emotional engagement is an important indicator of willingness prediction (Shuck et al., 2015). For example, the degree of school internship courses engagement positively affects intention to continue participating (Tseng and Chen, 2015). Other studies have also confirmed that the degree of participants’ engagement has positive impacts on creating value and the intention to participate (Algharabat, 2018). In Costley and Lange’s (2017) study on digital learning videos, learners’ engagement level positively affected their future behavioral willingness. The above literature shows that the level of engagement and continued participation are positively correlated with each other. The results of this study also show a positive correlation between three types of cram school engagement and the intention to continue attending cram school.

## Conclusion

Attending cram school has long been a trend in ethnic Chinese culture areas, including Taiwan. A high percentage of Taiwanese students have experience of their parents arranging for them to attend cram school during their school life. Since attending cram school is a universal routine, it is of great importance to understand cram school engagement status. Padilla-Walker and Nelson (2012) indicated that the more parents are involved in their children’s daily life, the less their children would engage in school. The results of this study show that hovering behavior awareness is negatively related to cram school engagement, whereas cram school engagement is positively related to the intention to continue attending cram school. This represents that helicopter parenting negatively affects the situation of learners’ cram school engagement as well as the intention to continue attending cram school, and this outcome affects the effectiveness of cram schooling.

### Implications

Parenting has great impacts on adolescent behaviors. In a society with a low birth rate such as Taiwan, parents are often overly involved in their children’s lives (Hong et al., 2015). Since studies have proved the negative impacts helicopter parenting has on learners and cram school education, what methods parents should adopt to nurture their children is a critical issue.

Studies have also shown that students’ learning situation can indeed be ameliorated by enhancing their engagement level (Bryson and Hand, 2007). Thus, both parents and cram school teachers, especially in regions where cram schooling culture prevails, should pay attention to learners’ engagement situation in school to help them maintain intention to continue attending cram school, in order to make cram schooling effective.

### Limitations and Future Research

Although this study validated the relationship between helicopter parenting and tutoring engagement, it is not known how tutoring engagement affect the real academic performance in their own schools. However, students attended English cram schools were from different levels of academic performance, it is difficult to evaluate the effect of learning achievement based on their school levels. Therefore, a follow-up study can be applied to further investigate the specific factors that attending English cram schools contribute to their effect of English learning on regular schools.

Learners’ knowledge growth in specific topics can be a predictive indicator of learning behavior (Carpenter et al., 2016). However, the relationship between learners’ cram school engagement and school behaviors was not discussed in this study. Research regarding this topic can be conducted in the future.

In addition, this was a confirmatory study. The hovering behavior awareness and cram school engagement of cram school attendees of different educational systems or ages were not covered in this study. However, this is also an important issue to be discussed in terms of cram school education. Thus, this topic could be further developed and analyzed in the upcoming studies.

## Data Availability Statement

The raw data supporting the conclusions of this article will be made available by the authors, without undue reservation.

## Ethics Statement

Ethical review and approval was not required for the study on human participants in accordance with the local legislation and institutional requirements. Written informed consent from the participants’ legal guardian/next of kin was not required to participate in this study in accordance with the national legislation and the institutional requirements.

## Author Contributions

Y-JH, J-CH, J-HY, P-HC, and Y-JCL: concept and design and drafting of the manuscript. J-CH, J-HY, and Y-JCL: acquisition of data and statistical analysis. P-HC and L-PM: critical revision of the manuscript. All authors contributed to the article and approved the submitted version.

## Conflict of Interest

The authors declare that the research was conducted in the absence of any commercial or financial relationships that could be construed as a potential conflict of interest.

## Publisher’s Note

All claims expressed in this article are solely those of the authors and do not necessarily represent those of their affiliated organizations, or those of the publisher, the editors and the reviewers. Any product that may be evaluated in this article, or claim that may be made by its manufacturer, is not guaranteed or endorsed by the publisher.

## Appendix

**TABLE A1:** Questionnaire.

Item	*M*	*SD*
**Life hovering**		
(1) My parents never ask me to do housework.	2.35	0.92
(2) When I go to school, my parents always carry my school bag for me.	2.19	0.89
(3) I seldom tidy my bedroom by myself; my parents always do it.	2.29	0.88
(4) My parents never ask me to do laundry.	2.21	0.87
(5) When I go to a friend’s house, even if it is close to my house, my parents will go with me.	2.49	0.88
(6) My parents always ask me to provide more information about my new friends.	2.29	0.89
**Coursework hovering**		
(1) If there are too many assignments from school, my parents will help me complete them.	1.91	0.93
(2) If there are some mistakes in a test that need to be revised, my parents will do it for me.	2.09	0.89
(3) Before a school test, my parents will tutor me, even if I had tutoring at cram school.	2.05	0.91
(4) If I forget to bring some things which I have to give to the teacher, my parents will leave work and take them to school for me.	2.25	0.87
(5) If I am punished at school, my parents will go to school to understand the reason and even blame the teachers.	2.04	0.92
**Behavioral engagement**		
(1) I usually attend cram school on time.	3.83	0.93
(2) I usually complete my assignments on time.	3.83	0.94
(3) No matter what kind of weather it is, I try to attend cram school on time.	3.63	0.95
(4) I usually do not fall asleep when I attend cram school.	3.70	0.98
**Emotional engagement**		
(1) I like to attend cram school after school.	3.33	0.83
(2) Even if I am tired from schoolwork, I feel comfortable when I get to cram school.	3.57	0.81
(3) I enjoy being tutored at cram school.	3.38	0.80
(4) I like to be tutored to master some concepts at cram school.	3.40	0.84
(5) Even if my performance has not improved, I still like the way of tutoring in cram school.	3.51	0.80
**Cognitive engagement**		
(1) I usually concentrate on what the tutors teach in cram school.	3.37	0.90
(2) I take notes when the tutors are teaching.	3.65	0.87
(3) I will mark those mistakes I made and practice them to avoid making the same errors again.	3.71	0.86
(4) I usually make a summary after the tutor’s lecture.	3.42	0.86
(5) I usually compare the similarities and differences in learning between what regular school teaches and the tutor teaches at cram school.	3.46	0.89
**Continued attendance**		
(1) I will attend cram school for tutoring no matter how useful it is for me.	3.66	0.84
(2) I will continue attending cram school even if my academic performance has not improved	3.60	0.89
(3) I will continue attending cram school to learn more than just the school homework.	3.73	0.88
(4) I will attend cram school no matter what else I have to do.	3.59	0.87
(5) If I do not like a tutor, I will change to another tutor, but will continue attending cram school.	3.64	0.89
